# Supplementary material for: MetaRibo-Seq measures translation in microbiomes
Source: Nat Commun. 2020 Jun 29;11:3268. doi: 10.1038/s41467-020-17081-z (PMC7324362; doi:10.1038/s41467-020-17081-z)
Supplement: Supplementary file 10 — Supplementary Data 7 [file 41467_2020_17081_MOESM10_ESM.zip › File2/Confidence_High_Taxonomy/244636_out.krona.html]

Javascript must be enabled to view this page.

members
magnitude
magnitudeUnassigned
count
unassigned
taxon
rank

244636\_out

3

3
2
superkingdom

3
976
phylum

class
3
200643

order
171549
3

family
2
2005525

genus
375288
2

1263094
1

SRS015782\_contig\_number\_33992
species

46503
1

SRS047044\_contig\_number\_5607
species

815
1
family

genus
816
1

species

SRS077231\_contig\_number\_18665
1
46506
